# Association between short-term radiation-induced toxicity and oncological outcomes in high-risk prostate cancer: a retrospective single-centre cohort study

**DOI:** 10.2340/1651-226X.2026.45030

**Published:** 2026-03-23

**Authors:** Jenny Kahlmeter Brandell, Antonis Valachis, Henrik Ugge, Daniel Robert Smith, Bengt Johansson

**Affiliations:** a Department of Oncology, Faculty of Medicine and Health, Örebro University Hospital, Örebro University, Örebro, Sweden; b Department of Urology, Faculty of Medicine and Health, Örebro University, Örebro, Sweden; c School of Medical Sciences, Örebro University, Örebro, Sweden; d Clinical Epidemiology and Biostatistics, School of Medical Sciences, Örebro University, Örebro, Sweden; e The Institute of Cancer Research, London, UK

**Keywords:** prostatic neoplasms, radiotherapy, treatment outcome, treatment-related side effects

## Abstract

**Background and purpose:**

This retrospective cohort study aimed to explore the association between short-term genitourinary (GU) and gastrointestinal (GI) toxicity and oncological outcomes in high-risk prostate cancer patients treated with external beam radiotherapy (EBRT) alone or with high-dose-rate brachytherapy (HDR-BT).

**Patient/material and methods:**

High-risk prostate cancer patients treated at Örebro University Hospital (2008–2021) were divided into two cohorts based on treatment modality: EBRT-only (66 Gy/22 fractions), EBRT-BT (42 Gy/14 fractions + 14.5 Gy HDR-BT-boost).

**Maximum 6-month toxicity grade was categorised as:**

GU-low (grade 0–1), GU-high (grade ≥ 2), GI-low (grade 0) and GI-high (grade ≥ 1), respectively. Freedom from biochemical failure (FFBF), metastasis-free survival (MFS) and overall survival were compared between the low- and high- toxicity groups, using Kaplan–Meier and Cox proportional hazards regression. Prostate cancer-specific mortality was compared between the groups using the Aalen–Johansen method and Fine-Gray regression.

**Results:**

The EBRT-only cohort encompassed 114 and 162 patients for GU- and GI-analyses. The EBRT-BT cohort comprised 306 patients for GU- and 344 patients for GI-analyses. High GU-toxicity was associated with inferior FFBF (adjusted hazard ratio (aHR) = 2.57, 95% Confidence Interval [CI] = 1.32–5.00) and MFS (aHR = 2.22, 95% CI = 1.21–4.07) in the EBRT-only but not in the EBRT-BT cohort. No statistically significant associations were found between GI-toxicity and oncological outcomes.

**Interpretation:**

Early grade ≥ 2 GU-toxicity was linked to worse FFBR and MFS after EBRT alone, whereas no such association was seen after EBRT-BT. GI toxicity showed no prognostic impact. These exploratory findings warrant validation in larger studies addressing interactions between patient-, tumour- and treatment-related factors.

## Introduction

Radiation therapy (RT) with or without androgen deprivation therapy (ADT) is a standard treatment for prostate cancer [1, 2]. Radiation affects adjacent normal tissues, causing treatment-related toxicity, which varies between patients [3, 4]. Acute and persistent genitourinary (GU) and gastrointestinal (GI) toxicity has been linked to late toxicity and diminished quality of life [5, 6], but its potential association with long-term oncological outcomes remains unclear. Furthermore, reliable surrogate markers for early assessment of long-term outcomes after RT in prostate cancer are lacking [7].

Previous studies suggest that worsening short-term treatment-related symptoms following prostate cancer radiation may be associated with poorer long-term oncological outcomes [8, 9]; however, the evidence is limited and primarily based on low- to intermediate-risk patients treated with conventionally fractionated external beam radiotherapy (EBRT), with or without short term ADT. High‑risk patients and other radiotherapy approaches have been less extensively studied in this context.

Therefore, this retrospective cohort study aimed to investigate the association between short-term GU- and GI-toxicity and oncological outcomes in high-risk prostate cancer patients treated with EBRT alone or in combination with high-dose-rate brachytherapy (HDR-BT). Improved understanding of these relationships may help to identify early factors worth examining in future research on long‑term outcomes and follow‑up.

## Patients/material and methods

### Study design and patient selection

This is a retrospective cohort study based on prospectively collected data. Patients were identified from an institutional database of all prostate cancer patients treated with RT at Örebro University Hospital from 2008 to 2021. Men with histologically verified high- or very high-risk prostate cancer, without lymph node or distant metastasis, curatively treated with moderately hypofractionated EBRT alone or combined with HDR-BT, were eligible. Risk group classification was adapted from National Comprehensive Cancer Network (NCCN) guidelines [1]. High-risk was defined as cT3, International Society of Urological Pathology (ISUP) grade group 4–5 [10], or prostate-specific antigen (PSA) ≥ 20 ng/mL. Patients were classified as very high-risk when meeting at least two of the criteria: cT3, ISUP grade group 4–5, or PSA > 40 ng/mL. Patients with cT4 or PSA > 100 ng/mL were not included. The patients were divided into two cohorts based on treatment modality: EBRT-only and EBRT-BT. As pre-specified in the study design, the two treatment cohorts were analysed separately to account for radiobiological differences between the treatment approaches. Per‑patient information on the rationale for selection of treatment strategy was not available. In clinical practice, however, selection was generally guided by prostate volume, local tumour extent, patient preference, and the availability of brachytherapy.

### Procedures

EBRT was delivered with daily image-guided (fiducial marker- or bone-based), three-dimensional conformal technique (3DCRT) from 2008 to 2013 and volumetric modulated arc therapy (VMAT) from 2014 onwards. The EBRT-only cohort received 66 Gy over 22 fractions (Equivalent dose in 2-Gy fractions [EQD2_α/β=3_] = 79 Gy), while the EBRT-BT-cohort underwent EBRT to 42 Gy along with a 14.5 Gy HDR-BT boost (EQD2_α/β=3_ = 101 Gy). Both cohorts received either prostate-only or additional elective pelvic nodal radiation therapy (PNRT) of 42 Gy in 14 fractions. All EBRT was delivered with 3 Gy per fraction, three times weekly.

For EBRT, the clinical target volumes (CTV) for prostate, seminal vesicles and pelvic lymph nodes (if included) were delineated on computed tomography (CT)-images. The extent of pelvic lymph node areas included in the CTV varied over time, as previously described [11]. The CTV to planning target volume (PTV) margin for prostate and seminal vesicles was 7 mm for patients with implanted fiducial markers and 10–15 mm for bone-based set-up, based on regional guidelines at the time. The CTV-PTV margin for pelvic lymph nodes was 5 mm. Organs at risk (bladder, rectum, bowel-bag and femoral heads) were delineated according to local guidelines. Dose constraints for EBRT were modified from QUANTEC [12], Fiorino et al. [13] and Widmark et al. [14].

For HDR-BT, prostate CTV was delineated using ultrasound. PTV equalled CTV. Urethra, rectal wall, and rectal mucosa were delineated 5 mm beyond CTV. The HDR-BT method and dose constraints are published elsewhere [15].

Most patients received anti-androgen therapy only (most often bicalutamide 150 mg orally for 24–36 months), according to local guidelines at the time.

### Follow-up

Patients were followed with a clinical visit at the end of RT, an oncology nurse contact at 3 weeks, there after every 6 months for 3 years and then annually for 10 years. PSA levels and toxicity were evaluated at each follow-up. GU- and GI-toxicities were assessed by a trained nurse or physician, using a local grading scale (Supplementary Appendix A, Table A.1), modified from the Radiation Therapy Oncology Group (RTOG) grading scale [16], based on a telephone contact or standardised questionnaires. Radiological assessments were carried out based on clinical indication.

### Data collection

Data on patient- and tumour characteristics, treatment-related parameters, outcomes and toxicity were extracted from the database. Tumour characteristics, outcome measures and treatment parameters were subsequently verified and supplemented during review of patient records and RT systems, without knowledge of individual toxicity-grades. Data were collected between 1st January 2008 and 30th September 2023.

### Definitions and outcome measures

Patients in each cohort were divided into two groups based on their maximum 6-month toxicity, defined as the highest observed toxicity grade measured at 3 weeks and 6 months, and categorised as follows: GU-low (grade 0–1), GU-high (grade ≥ 2), GI-low (grade 0) and GI-high (grade ≥ 1), respectively. The cut-off for GI-high was set to ≥1 due to the low frequency of grade ≥ 2 GI-toxicity. Patients with missing toxicity-data at one time-point were included in the analysis. Patients with Grade ≥ 2 or unknown baseline GU-symptom grade were excluded from GU-analyses. Information on baseline GI function was not available. Primary and secondary endpoints were compared between the low- and high- toxicity groups (GU-low versus GU-high and GI-low versus GI-high).

*Primary endpoint* was freedom from biochemical failure (FFBF), defined according to the Phoenix criteria [17] (PSA nadir + 2 ng/mL). Missing PSA-values (gaps in follow-up) were carefully revised through review of patient’s record. If there were no signs of relapse during the gap, the last available observation was carried forward. Patients who did not experience biochemical failure, including those who died before any failure occurred, were censored at the date of their most recent PSA test.

*Secondary endpoints* included metastasis-free survival (MFS), defined as the absence of regional or distant metastasis, or death; overall survival (OS); and prostate cancer-specific mortality (PCSM), defined as death attributed to prostate cancer. Cause of death was determined from patient records. If unknown, patients with metastatic castration-resistant prostate cancer were classified as having died from prostate cancer, while those without recurrence or with stable hormone-sensitive disease were categorised as having died from other causes.

Metastasis-free patients were censored at the latest review of their patient records (for MFS) and surviving patients at the last population registry cross-check (for OS and PCSM). Follow-up for analysis of time-to-event outcomes was initiated upon completion of RT.

### Statistical analysis

Patient-, tumour- and treatment characteristics were summarised using descriptive statistics. Median follow-up was calculated using reversed Kaplan–Meier methods.

The Kaplan–Meier method was applied for estimating FFBF, MFS and OS and survival rates compared via log-rank test. Crude and adjusted hazard ratios (aHRs) along with 95% confidence intervals (95% CI) were estimated using Cox proportional hazards regression models. PCSM was estimated using the Aalen–Johansen method, treating other-cause mortality as a competing event. Group comparisons were performed using Gray’s test, and subdistribution hazard ratios (HRs), along with 95% CI, were obtained from Fine–Gray regression.

In the main analyses, the multivariable models included maximum 6-month toxicity (low versus high), age (as continuous variable), T-stage (1–2 versus 3) and radiation technique (3DCRT versus VMAT). Across the separate analyses, the number of events ranged from 37 to 72 for the primary endpoint. With this in mind we carefully pre-specified variables for covariate adjustment to account for confounding, while being mindful of overfitting.

To explore the robustness of primary analyses, we performed a sensitivity analysis, using an alternative Cox-model, including age, T-stage, ISUP-grade and PNRT. Missing ISUP‑grade values were imputed using the most frequent category (modal imputation). Additional sensitivity analyses were conducted to examine the relationship between baseline GU-symptom grade (0 versus 1) and the primary outcome. If significant associations were identified, further subgroup analyses were performed, stratified by baseline GU-symptom grade, to assess the link between maximum 6-month toxicity and the primary endpoint within each subgroup. Since the exposure (maximum 6-month toxicity) occurred during the first 6 months of follow-up an extra sensitivity analysis was carried out, excluding patients with time-in-study shorter than 6 months. Competing risk analyses were conducted to estimate the cumulative incidence of metastases, using the Aalen–Johansen method while treating death as a competing event. Comparisons were performed using Gray’s test and subdistribution hazards were estimated with Fine-Gray regression.

Proportional hazards were evaluated using Schoenfeld residuals (*p*-values and plots) for the Cox models and time‑dependent interaction terms for the Fine–Gray models. No systematic model‑level violations were observed; minor variable‑level deviations were inconsistent and expected given the limited number of events. In these cases, robust (sandwich) variance estimators were applied to obtain consistent standard errors, and the HRs were interpreted as time‑averaged effects over follow‑up. A *p*-value below 0.05 was regarded as statistically significant. All analyses were performed in R version 4.3.2.

## Results

### Study population

**Figure 1:**
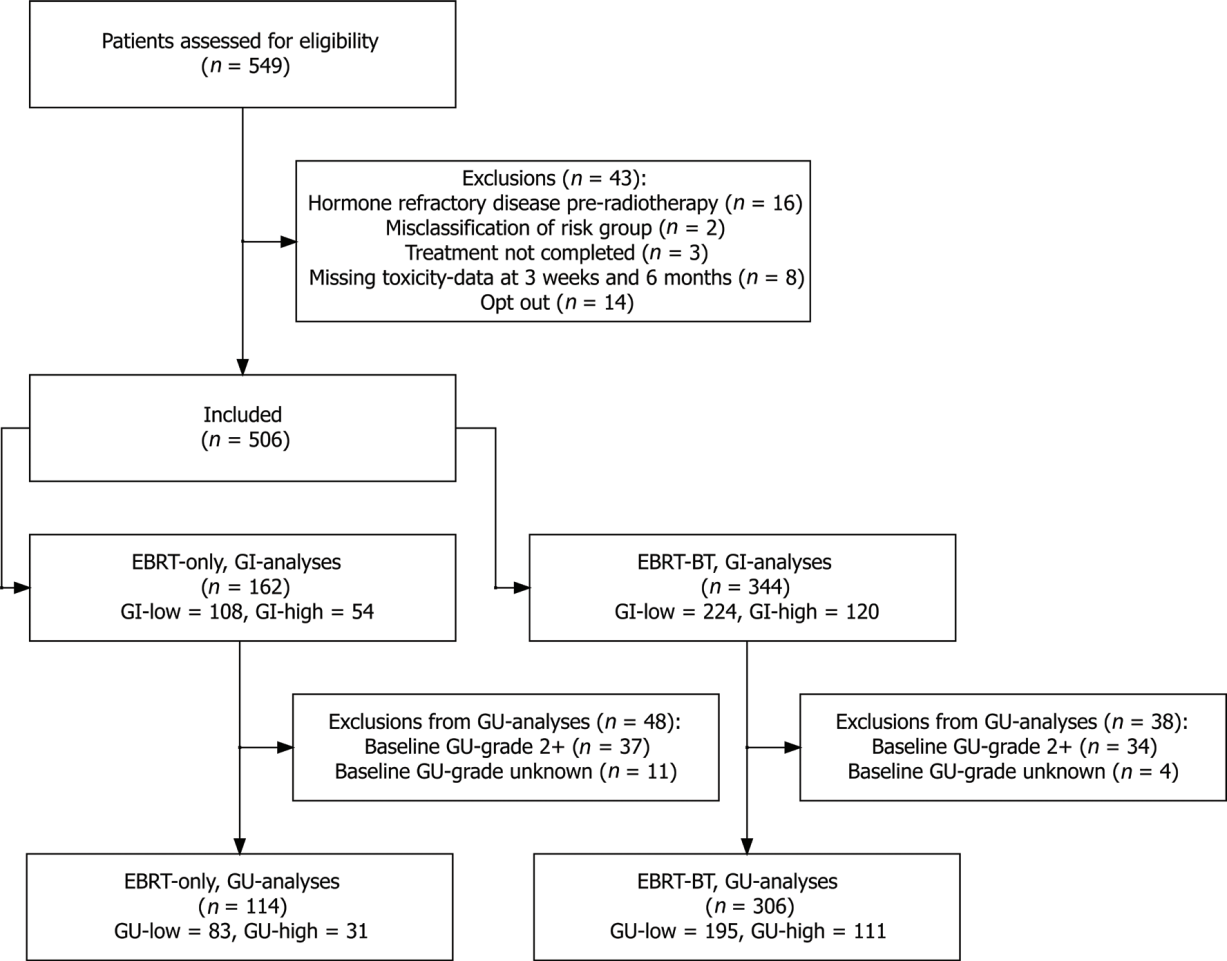
Flowchart of cohorts and exclusions. EBRT: external beam radiotherapy; BT: brachytherapy; GI: gastrointestinal; GU: genitourinary.

A total of 549 eligible patients was identified as the source population. After applying the predefined exclusion criteria (summarised in Figure 1), the study population was divided into the two treatment cohorts: EBRT‑only and EBRT‑BT.

For GU analyses, after exclusion of patients with Grade ≥ 2 or unknown baseline GU-symptom grade, the EBRT‑only cohort included 114 patients (83 low‑ and 31 high‑toxicity), while the EBRT‑BT cohort included 306 patients (195 low‑ and 111 high‑toxicity). For GI analyses, the EBRT‑only cohort comprised 162 patients (108 low‑ and 54 high‑toxicity), and the EBRT‑BT cohort comprised 344 patients (224 low‑ and 120 high‑toxicity).

Baseline characteristics for GU-analyses are summarised in Table 1 and for GI-analyses in Supplementary Appendix A (Table A.2–A.3). Within each treatment cohort, the low‑ and high‑toxicity groups were generally comparable in age, clinical risk factors, and treatment-related parameters. The main exceptions were that within the EBRT‑BT cohort, GU high‑toxicity patients were slightly older, and very‑high‑risk status was more common in the GI low‑toxicity group than in the GI high-toxicity group.

**Table 1:** Baseline characteristics for GU-analyses in the EBRT-only (A) and EBRT-BT (B) cohort.

Variable	A. EBRT-only cohort Maximum 6-month GU toxicity			B. EBRT-BT cohort Maximum 6-month GU toxicity		
	Total, *N* = 114^1^	GU-low, *N* = 83^1^	GU-high, *N* = 31^1^	Total, *N* = 306^1^	GU-low, *N* = 195^1^	GU-high, *N* = 111^1^
Baseline GU-grade						
0	61 (54%)	52 (63%)	9 (29%)	182 (59%)	134 (69%)	48 (43%)
1	53 (46%)	31 (37%)	22 (71%)	124 (41%)	61 (31%)	63 (57%)
**Age at diagnosis (years)**	69 (67, 74)	69 (67, 74)	69 (67, 73)	70 (66, 74)	69 (66, 73)	71 (68, 74)
**Year of treatment**	2013 (2011, 2017)	2013 (2010, 2017)	2013 (2011, 2016)	2016 (2013, 2018)	2015 (2013, 2018)	2016 (2013, 2018)
**PSA (ng/mL)**	20 (11, 32)	20 (12, 32)	18 (11, 31)	19 (9, 30)	19 (8, 31)	18 (9, 30)
**ISUP grade group** ^2^						
Group ≤ 3	41 (36%)	29 (35%)	12 (39%)	136 (44%)	86 (44%)	50 (45%)
Group 4	33 (29%)	24 (29%)	9 (29%)	86 (28%)	54 (28%)	32 (29%)
Group 5	37 (32%)	28 (34%)	9 (29%)	84 (27%)	55 (28%)	29 (26%)
Unknown	3 (2.6%)	2 (2.4%)	1 (3.2%)	0 (0%)	0 (0%)	0 (0%)
**Clinical T-stage**						
1	31 (27%)	22 (27%)	9 (29%)	82 (27%)	55 (28%)	27 (24%)
2	32 (28%)	24 (29%)	8 (26%)	90 (29%)	60 (31%)	30 (27%)
3	51 (45%)	37 (45%)	14 (45%)	134 (44%)	80 (41%)	54 (49%)
**Number of high-risk factors**						
1	60 (53%)	41 (49%)	19 (61%)	186 (61%)	122 (63%)	64 (58%)
2	42 (37%)	33 (40%)	9 (29%)	91 (30%)	56 (29%)	35 (32%)
3	12 (11%)	9 (11%)	3 (9.7%)	29 (9.5%)	17 (8.7%)	12 (11%)
**Risk group** ^3^						
High	72 (63%)	51 (61%)	21 (68%)	227 (74%)	145 (74%)	82 (74%)
Very high	42 (37%)	32 (39%)	10 (32%)	79 (26%)	50 (26%)	29 (26%)
**Prostate volume (cc)**	51 (35, 66)	52 (40, 65)	50 (33, 66)	36 (29, 45)	35 (28, 45)	38 (30, 45)
Unknown	7	7	0	4	2	2
**Elective PNRT**	74 (65%)	54 (65%)	20 (65%)	163 (53%)	103 (53%)	60 (54%)
**Seminal vesicles included**	103 (90%)	74 (89%)	29 (94%)	272 (89%)	178 (91%)	94 (85%)
**Radiation technique**						
3DCRT	65 (57%)	48 (58%)	17 (55%)	92 (30%)	62 (32%)	30 (27%)
VMAT	49 (43%)	35 (42%)	14 (45%)	214 (70%)	133 (68%)	81 (73%)
**Fiducials**	105 (92%)	76 (92%)	29 (94%)	55 (18%)	41 (21%)	14 (13%)
**Antihormonal therapy**						
None	4 (3.5%)	2 (2.4%)	2 (6.5%)	13 (4.2%)	9 (4.6%)	4 (3.6%)
Antiandrogen only	100 (88%)	73 (88%)	27 (87%)	251 (82%)	159 (82%)	92 (83%)
ADT or CAB	10 (8.8%)	8 (9.6%)	2 (6.5%)	42 (14%)	27 (14%)	15 (14%)
**Duration of antihormonal therapy (months)**	29 (27, 30)	29 (27, 30)	29 (28, 30)	28 (27, 29)	28 (27, 29)	28 (28, 29)

EBRT: external beam radiotherapy; BT: brachytherapy; GU: genitourinary; PSA: prostate-specific antigen; PNRT: pelvic nodal radiation therapy; 3DCRT: three-dimensional conformal radiation therapy; VMAT: volumetric modulated arc therapy; ADT: androgen deprivation therapy; CAB: combined androgen blockade, i.e. first generation antiandrogen in combination with gonadotropin-releasing hormone-agonist or antagonist.

1 Median (IQR) or Frequency (%).

2 According to the 2014 International Society of Urological Pathology (ISUP) Consensus Conference on Gleason Grading of Prostatic Carcinoma [9].

3 Risk group classification adapted from Comprehensive Cancer Network (NCCN) guidelines [1].

Table 1 also provides a between‑cohort comparison, indicating that the two treatment cohorts were broadly similar. The main differences were that the EBRT‑only cohort had larger prostate volumes, was treated earlier, had fewer patients receiving VMAT, and included a slightly higher proportion of very high‑risk patients. The prevalence of T3 tumours was comparable between cohorts; however, information on T3 substages (T3a versus T3b) was unavailable.

### Association between early toxicity and oncologic outcomes

Across all analyses, the clearest pattern emerged in the EBRT only cohort, where high maximum 6-month GU toxicity correlated with inferior oncologic outcomes. No such associations were observed in the EBRT-BT cohort. Median follow-up for all endpoints ranged from 6 to 11 years, with no differences between the toxicity groups (Table 2).

**Table 2:** A summary of statistics for GU- and GI-toxicity analyses in the EBRT-only and EBRT-BT cohort.

A.		GU-toxicity			
		EBRT-only		EBRT-BT	
Endpoint	Metric	GU-low	GU-high	GU-low	GU-high
	*N* (%)	83 (72.8%)	31 (27.2%)	195 (63.7%)	111 (36.3%)
FFBF	No. of events	21	16	38	26
	Median FU (95% CI, years)	8.24 (7.13–9.79)	8.89 (5.88–9.95)	6.11 (5.02–7.05)	5.99 (5.47–6.65)
	5-year FFBF (95% CI, %)	78.2 (69.0–88.7)	59.2 (43.1–81.4)	79.1 (72.8–85.9)	76.8 (68.7–85.9)
	aHR (95% CI)		**2.57 (1.32–5.00)**		1.05 (0.63–1.75)
MFS	No. of events	27	18	53	38
	Median FU (95% CI, years)	8.86 (7.91–10.27)	9.26 (6.61–11.33)	6.82 (5.47–7.61)	6.51 (6.10–7.76)
	5-year MFS (95% CI, %)	79.0 (70.3–88.7)	62.7 (47.4–82.9)	80.1 (74.3–86.4)	84.5 (77.7–91.8)
	aHR (95% CI)		**2.22 (1.21–4.07)**		1.17 (0.77–1.79)
OS	No. of events	22	12	37	28
	Median FU (95% CI, years)	10.79 (9.15–11.93)	9.80 (7.33–11.93)	7.54 (6.31–8.15)	7.75 (6.97–8.52)
	5-year OS (95% CI, %)	88.3 (81.4–95.8)	83.4 (71.1–97.8)	88.6 (84.0–93.5)	94.3 (89.9–98.8)
	aHR (95% CI)		1.74 (0.85–3.54)		1.23 (0.74–2.05)
PCSM	No. of events	11	7	12	10
	Median FU (95% CI, years)	9.42 (8.49–11.60)	9.69 (6.54–10.82)	6.77 (5.90–7.87)	7.18 (6.46–7.88)
	5-year cumulative PCSM (95% CI, %)	2.7 (0–6.4)	10.0 (0–21.0)	4.4 (1.2–7.5)	3.9 (1.2–7.7)
	aSHR (95% CI)		2.35 (0.90–6.15)		1.25 (0.55–2.81)

B.		GI-toxicity			
		EBRT-only		EBRT-BT	
Endpoint	Metric	GI-low	GI-high	GI-low	GI-high
	*N* (%)	108 (66.7%)	54 (33.3%)	224 (65.1%)	120 (34.9%)

FFBF	No. of events	34	24	46	26
	Median FU (95% CI, years)	8.22 (7.12–9.85)	8.99 (5.76–10.01)	5.94 (5.02–6.78)	5.92 (5.02–6.92)
	5-year FFBF (95% CI, %)	72.4 (63.9–82.1)	65.4 (52.8–81.0)	78.1 (72.2–84.6)	76.9 (68.9–85.9)
	aHR (95% CI)		1.52 (0.89–2.59)		1.11 (0.68–1.79)
MFS	No. of events	48	26	66	37
	Median FU (95% CI, years)	8.68 (7.91–10.27)	9.36 (5.79–10.45)	6.50 (5.77–7.24)	6.18 (5.46–7.30)
	5-year MFS (95% CI, %)	72.4 (64.2–81.7)	75.9 (64.8–88.9)	80.8 (75.5–86.6)	78.8 (71.4–87.0)
	aHR (95% CI)		1.3 (0.80–2.09)		1.13 (0.75–1.69)
OS	No. of events	38	18	49	26
	Median FU (95% CI, years)	11.33 (10.17–11.93)	10.07 (7.33–11.93)	7.50 (6.52–8.02)	6.97 (6.22–7.95)
	5-year OS (95% CI, %)	84.5 (77.7–91.8)	88.0 (79.4–97.5)	90.1 (86.1–94.4)	88.0 (82.1–94.4)
	aHR (95% CI)		1.12 (0.65–1.96)		1.03 (0.64–1.67
PCSM	No. of events	17	11	16	10
	Median FU (95% CI, years)	9.80 (8.51–10.85)	9.69 (6.54–10.31)	6.90 (6.28–7.75)	6.41 (6.03–7.33)
	5-year cumulative PCSM (95% CI, %)	5.9 (1.2–10.4)	10.1 (1.6–18.7)	3.7 (1.0–6.4)	5.6 (1.2–10.0)
	aSHR (95% CI)		1.50 (0.70–3.21)		1.23 (0.56–2.69)

Number of events, median follow‑up (estimated using the reversed Kaplan–Meier method), 5‑year estimates and adjusted effect measures for FFBF, MFS, OS and PCSM. FFBF, MFS and OS were analysed using Kaplan–Meier estimates and multivariable Cox proportional hazards models. PCSM reflects cumulative incidence, estimated using the Aalen–Johansen method with other‑cause mortality as a competing event; group comparisons were made using Gray’s test and subdistribution hazard ratios were obtained with Fine–Gray regression. The same covariates (toxicity grade, age, T‑stage, and radiation technique) were included in both models.

Results are shown separately for GU (A) and GI (B) toxicity in the EBRT‑only and EBRT‑BT cohorts, grouped by maximum 6‑month toxicity grade.

GU: genitourinary; GI: gastrointestinal; EBRT: external beam radiotherapy; BT: brachytherapy; FU: follow-up; FFBF: freedom from biochemical failure; MFS: metastasis-free survival; PCSM: prostate cancer-specific mortality; OS: overall survival; aHR: adjusted hazard ratio; aSHR: adjusted subdistribution hazard ratio.

#### Freedom from biochemical failure

**Figure 2:**
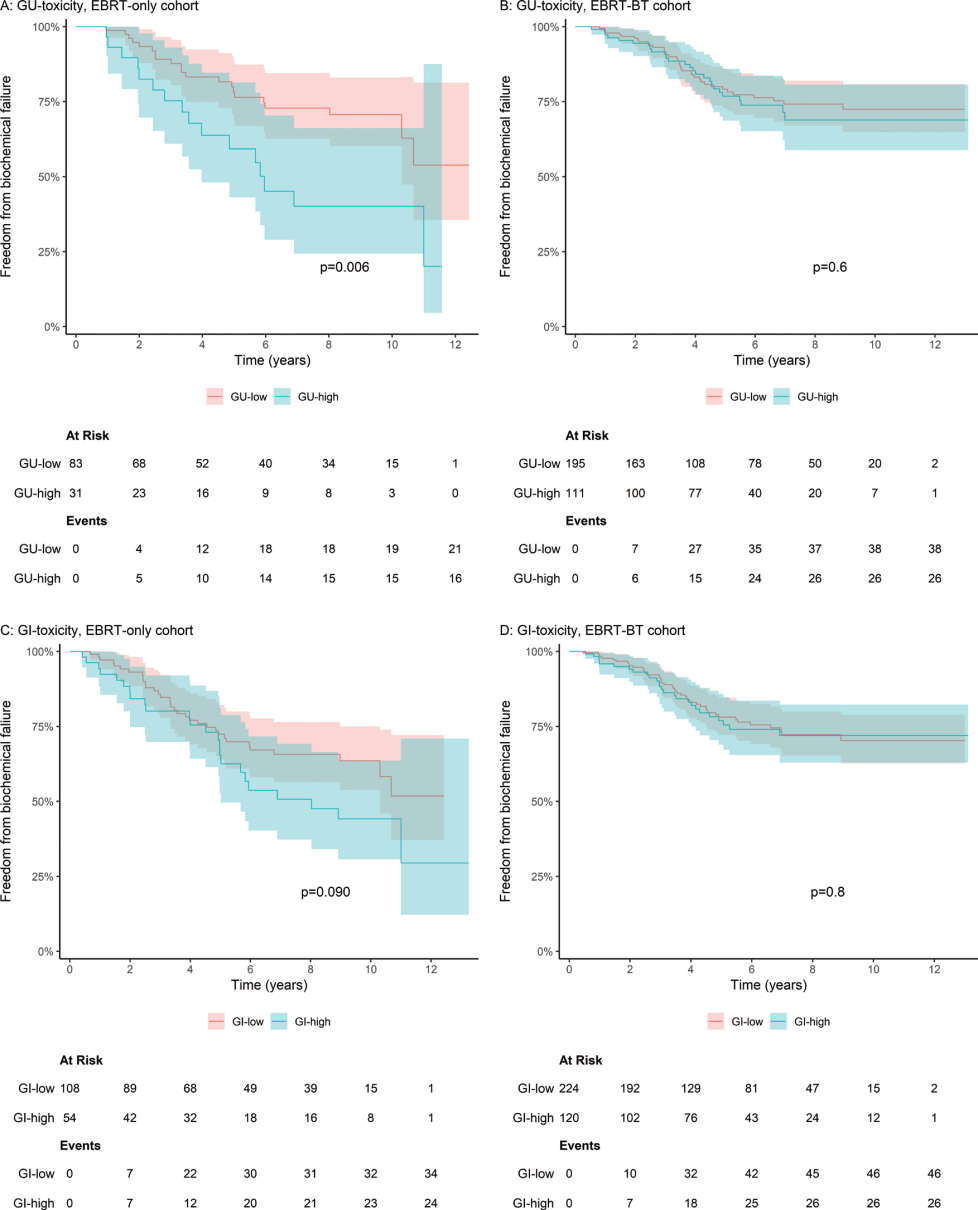
Freedom from biochemical failure, low versus high GU- och GI-toxicity in the EBRT-only and EBRT-BT cohort. Kaplan–Meier estimates of freedom from biochemical failure, with 95% confidence intervals, grouped by maximum 6-month GU- and GI-toxicity (low versus high): (A) GU-toxicity in the EBRT-only cohort, (B) GU-toxicity in the EBRT-BT cohort, (C) GI-toxicity in the EBRT-only cohort, (D) GI-toxicity in the EBRT-BT cohort. *P*-value for difference between survival curves computed using the log-rank test. GU: genitourinary; GI: gastrointestinal; EBRT: external beam radiotherapy; BT: brachytherapy.

In the EBRT-only cohort, patients with high GU toxicity had substantially lower unadjusted 5‑year FFBF rates than those with low toxicity (59% versus 78%, Figure 2). This association remained significant in both crude (HR 2.41, 95% CI 1.26–4.63) and adjusted models (aHR 2.57, 95% CI 1.32–5.00). No statistically significant association was found for GI toxicity (Figure 2 and Table 2).

In the EBRT-BT cohort, FFBF rates were similar between toxicity groups for both GU (77% versus 79%) and GI toxicity (78% versus 77%), with no statistically significant associations in crude or adjusted analyses (Figure 2 and Table 2).

#### Metastasis‑free survival, overall survival, and prostate cancer-specific mortality

**Figure 3:**
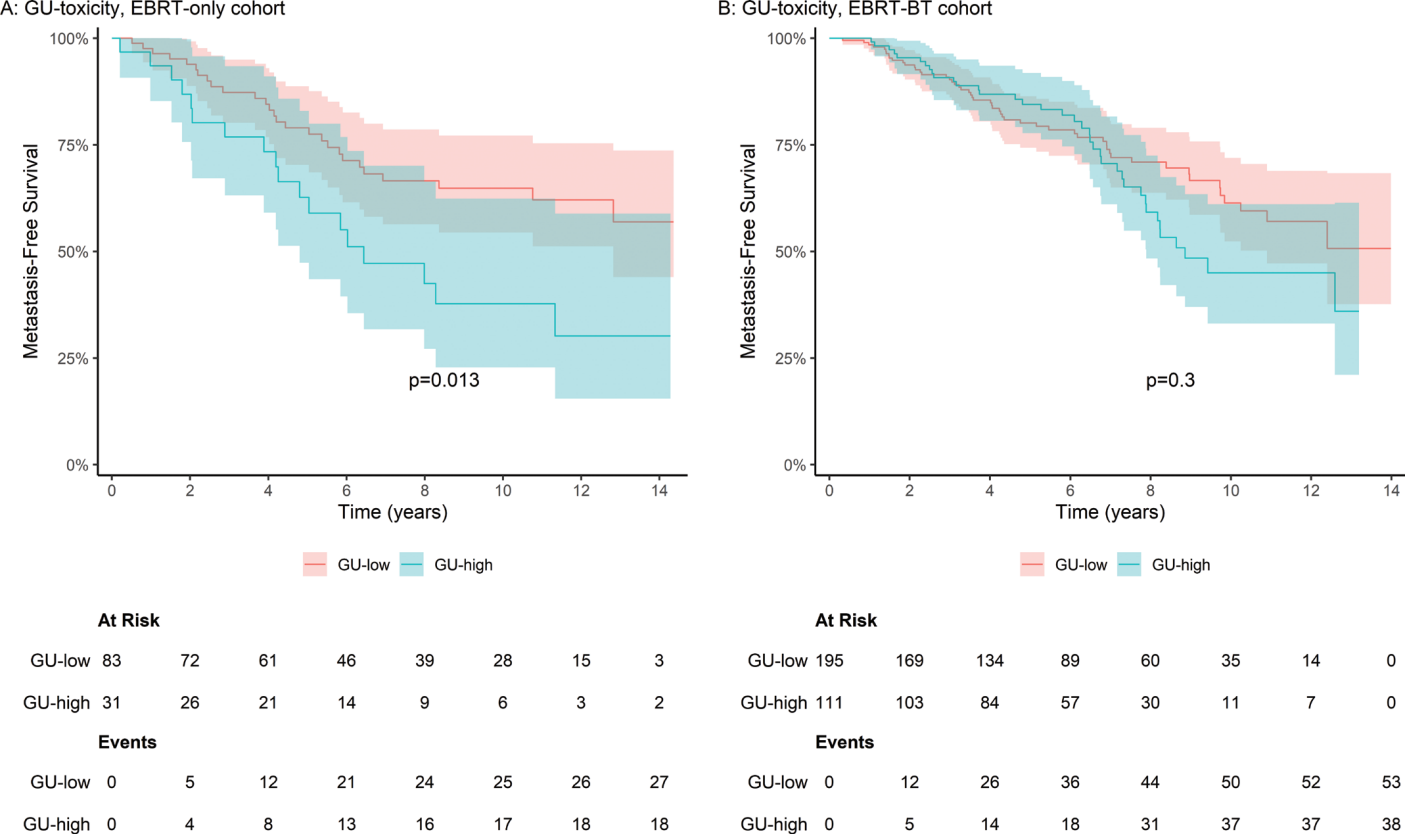
Metastasis-free survival, low versus high GU-toxicity in the EBRT-only and EBRT-BT cohort. Kaplan–Meier estimates of metastasis-free survival, with 95% confidence intervals, grouped by maximum 6-month GU-toxicity (low versus high), in the EBRT-only (A) and EBRT-BT (B) cohort. *P*-value for difference between survival curves computed using the log-rank test. GU: genitourinary; EBRT: external beam radiotherapy; BT: brachytherapy.

A similar pattern was observed for MFS, mirroring the findings for FFBF (Figure 3 and Table 2). In the EBRT‑only cohort, high GU toxicity was associated with reduced 5‑year MFS (63% versus 79%) and remained significant in adjusted analysis (aHR 2.22, 95% CI 1.21–4.07). No such association was found in the EBRT‑BT cohort (aHR 1.17, 95% CI 0.77–1.79). There were no statistically significant associations between early GU- or GI-toxicity and OS or PCSM in either cohort (Table 2 and Supplementary Appendix A, Figure A.2).

#### Additional covariate associations

In the primary analyses, T3 disease was associated with inferior FFBF in the EBRT-only cohort, whereas the use of VMAT showed a similar association in the EBRT-BT cohort (Supplementary Appendix A, Tables A.6 and A.11).

#### Sensitivity analyses

The robustness of the main analyses was generally supported by sensitivity analyses. Alternative Cox models (Supplementary Appendix A, Tables A.6–A.17) yielded similar estimates, and excluding patients with < 6 months follow‑up did not affect the results (not shown).

In the sensitivity analysis of the EBRT-BT cohort, baseline GU-symptom grade 1 was associated with inferior FFBF (aHR = 2.34, 95% CI = 1.41–3.90). However, patients with baseline grade 0 and 1 symptoms differed on important baseline characteristics (Supplementary Appendix A, Table A.5). Further subgroup analyses stratified by baseline GU-symptom grade showed no significant association between maximum 6-month GU-toxicity and FFBF in either subgroup (Supplementary Appendix A, Table A.14–A.15). There was no statistically significant association between baseline GU-symptom grade and FFBF in the EBRT-only cohort (aHR = 1.51, 95% CI = 0.75–3.03). In this cohort, patients with baseline grade 0 and grade 1 GU symptoms were largely similar, although grade 1 patients were treated later and more frequently received VMAT (Supplementary Appendix A, Table A.4).

Competing risk analyses for MFS yielded results consistent with the Kaplan–Meier estimates, indicating robustness across methods (Supplementary Appendix A, Table A.18–A.21, Figure A.1–A.2).

## Discussion and conclusion

In this retrospective study of two separate treatment cohorts of high- and very high-risk prostate cancer, we observed a statistically significant association between short-term grade ≥ 2 GU-toxicity and inferior FFBF and MFS in patients treated with moderately hypofractionated EBRT alone. In contrast, no such association was found in the cohort treated with combined EBRT and HDR-BT. These results suggest that a potential link between early GU toxicity and long-term outcome may be specific to the EBRT-only setting. However, although measured baseline variables were comparable between the treatment cohorts, unmeasured clinical considerations may still have guided treatment decisions and could partly explain the observed differences in outcomes between the two cohorts.

Our findings align with previous results [8, 9]. Vesprini et al. [8] demonstrated an association between grade ≥ 2 GU- and GI-toxicity and inferior biochemical control in prostate cancer patients treated with image guided EBRT. Similarly, Roy et al. [9] reported that short-term deterioration in patient-reported urinary symptoms was associated with inferior long-term event-free survival and MFS among patients with localised prostate cancer treated with EBRT alone, whereas no association was found between bowel symptoms and oncological outcomes.

The observed association of GU-toxicity with inferior oncological outcomes for patients treated with EBRT-only, as seen in our study and previous research, could be attributed to residual confounding, treatment-related uncertainties, or an underlying biological mechanism linking toxicity to RT response. Despite adjustment for known confounders, residual confounding may persist. Tumour characteristics not captured in the dataset, including tumour size, number of positive biopsy cores, extent of high Gleason score areas and the presence of extraprostatic extension and seminal vesicle involvement may have influenced treatment selection and oncological outcomes [18–21]. Their potential association with the risk of treatment-related toxicity, however, has been less extensively studied [19, 22]. Data on performance status, comorbidities, and smoking habits were also incomplete and may have confounded the results [23–26]. Although smoking data were unavailable in this study, the Swedish Public Health Agency reported a national daily smoking prevalence of 5–12% during the treatment period, suggesting a likely low proportion of daily smokers in the study population.

The results may also be confounded by baseline GU function. To mitigate this, patients with baseline grade ≥ 2 GU symptoms were excluded, increasing the likelihood that the analysed GU toxicity reflects treatment‑induced effects rather than pre‑existing urinary dysfunction. This design element reduces – but does not entirely eliminates – the risk that observed associations in the EBRT-only cohort were driven by baseline differences. Sensitivity analyses, which showed no clear association between baseline GU symptoms and oncologic outcomes, further support this interpretation.

In addition to residual confounding from patient- and tumour-related factors, uncertainties in treatment may have contributed to the observed associations. Variation in the dose delivered to the prostate and surrounding tissue may arise due to uncertainties in target definition, set-up errors, and intra- or inter-fractional organ motion or deformation [27–29], potentially affecting both long-term efficacy and toxicity [30]. Although geometric misses cannot be excluded, the risk is likely low due to the use of daily image guidance and PTV margins in the upper end of recommended ranges [31–34]. However, the image‑guided radiotherapy (IGRT) technology used did not include cone beam CT and real-time tracking or triggered imaging was not employed, slightly limiting precision compared with current standards. Additionally, the lack of magnetic resonance imaging (MRI) increases uncertainties in target definition, although CT-derived prostate volumes are generally larger than those obtained from MRI [35, 36].

Beyond residual confounding and treatment-related uncertainties, our findings may also reflect a biological mechanism, whereby acute GU toxicity arises from inflammation in the bladder, urethra, and prostate [37], potentially linked to radioresistance [38–40]. Elevated cytokine levels during and after radiotherapy have been associated with acute toxicity [41, 42]. Interleukin-6 (IL-6) may promote radioresistance by upregulating DNA-damage-repair-related molecules, such as breast cancer gene (BRCA1/2), ataxia telangiectasia mutated (ATM) and ataxia telangiectasia and Rad3-related (ATR) [39] and supporting tumour regrowth through RT-induced infiltration of myeloid-derived suppressor cells and angiogenesis [40]. Furthermore, RT can modulate the tumour microenvironment by activating cancer-associated fibroblasts and inducing recruitment and polarisation of tumour-associated macrophages through cytokine and complement signalling, which promotes an immunosuppressive and radioresistant phenotype in prostate tissue [43, 44]. While these fibro-inflammatory changes primarily support tumour survival, they may also influence acute toxicity in the surrounding normal tissues, though the direction and magnitude of this effect remain uncertain.

In this context, the observed difference in results between the two treatment cohorts may be attributable to differences in both the distinct effects of the treatment regimens on immune response, tumour stroma, and the tumour microenvironment [45], as well as differences in biological dose. If a true relationship exists between toxicity and radioresistance, the higher dose in the combined treatment could potentially overcome this effect.

In the EBRT–BT cohort, the use of VMAT technique was unexpectedly associated with inferior oncologic outcomes. This finding lacks a clear biological or technical explanation and should therefore be interpreted with caution. Given the absence of an established mechanistic rationale and the potential for residual confounding or chance findings, this observation is considered hypothesis-generating and warrants further investigation in independent cohorts.

The strengths of our study include consecutive follow-up with prospective toxicity grading and outcome registration, enhancing data reliability. In addition, the thorough verification of tumour-, outcome-, and treatment-related variables further improves data accuracy. The consistency and robustness of our results across multiple analyses, reinforce the validity of our findings.

Beyond previously discussed limitations related to residual confounding and treatment uncertainties, additional concerns remain. The relatively small number of events in the EBRT-only cohort may have limited the statistical precision. Because death was not coded as a competing event in the FFBF definition, competing risks could not be accounted for, potentially biasing the Kaplan–Meier estimates. The absence of baseline GI symptom grading could confound the GI-analyses, as it prevents differentiation between treatment-induced GI-toxicity and pre-existing symptoms. Moreover, information on dose levels to organs at risk was not available, limiting the ability to relate toxicity outcomes to actual dose distributions. Finally, the external validity of our results may be limited, as all patients were treated at a single institution using specific radiotherapeutic techniques and dose-fractionation schemes.

In conclusion, our study demonstrated a statistically significant association between grade ≥ 2 maximum 6-month GU-toxicity and inferior long-term oncologic outcomes in high-risk prostate cancer patients treated with EBRT alone, but not in those receiving combined EBRT and HDR-BT. No associations between GI-toxicities and oncological outcomes were observed. Larger studies employing modern RT techniques and investigating potential interactions among patient-related factors, tumour biology, and treatment modality are needed to validate and further explore our findings. If early GU toxicity proves to be a reliable marker of poorer outcome, it may help tailor treatment and follow-up on an individual basis.

## Supplementary Material



## Data Availability

The datasets analysed during this study are available from the corresponding author on reasonable request.
