# Supplementary material for: Association between short-term radiation-induced toxicity and oncological outcomes in high-risk prostate cancer: a retrospective single-centre cohort study
Source: Acta Oncol. 2026 Mar 23;65:45030. doi: 10.2340/1651-226X.2026.45030 (PMC13019436; doi:10.2340/1651-226X.2026.45030)
Supplement: Supplementary file 1 [file AO-65-45030-s1.pdf]

Appendix A

Table of Contents

Table A.1. Toxicity grading scale 2

Table A.2. Baseline characteristics for the EBRT-only cohort, GI-analyses........ 3

Table A.3. Baseline characteristics for the EBRT-BT cohort, GI-analyses 5

Table A.4. Baseline characteristics for the EBRT-only cohort, grouped by baseline GU-symptom grade........ 7

Table A.5. Baseline characteristics for the EBRT-BT cohort, grouped by baseline GU-symptom grade........ 9

Table A.6-A.8. Association between FFBF and GU-toxicity in the EBRT-only cohort 11

Table A.9-A.10. Association between FFBF and GI-toxicity in the EBRT-only cohort........ 13

Table A.11-A.15. Association between FFBF and GU-toxicity in the EBRT-BT cohort 14

Table A.16-A.17. Association between FFBF and GI-toxicity in the EBRT-BT cohort........ 17

Table A.18-A.19. Association between MFS and GU-toxicity in the EBRT-only cohort........ 18

Table A.20. Association between PCSM and GU-toxicity in the EBRT-only cohort 19

Figure A.1 Estimated cumulative incidence of metastatic disease, low versus high GU-toxicity grade, in the EBRT-only and EBRT-BT cohort 20

Figure A.2 Estimated cumulative incidence of prostate-cancer specific death, low versus high GU-toxicity grade, in the EBRT-only and EBRT-BT cohort 21

References........ 22

Table A.1. Toxicity grading scale

	**Grade 0**	**Grade 1**	**Grade 2**	**Grade 3**	**Grade 4**
**Genitourinary (GU)**	0-1 nocturia, no dysuria or urgency	2-3 nocturia, single dysuria or urgency	3-4 nocturia, sporadic dysuria or urgency, or need of medical treatment	> 4 nocturia, severe urgency or need for temporary catheter	Need for permanent catheter
**Gastrointestinal (GI)**	1-2 daily defecation, no urgency	3-4 daily defecations, single urgency or bloody stool	> 4 daily defecations, frequent urgency or bloody stool, need of medication	Continuous problems or need for surgical treatment	
**Erectile Dysfunction (ED)**	Well-functioning erection	Weak or non-maintained erection	No erection		
**Sexual life (SL)**	Satisfying sexual life	Impaired sexual life	No sexual life		

Toxicity grading scale, locally modified from Radiation Therapy Oncology Group (RTOG) grading scale [1].

Table A.2. Baseline characteristics for the EBRT-only cohort, GI-analyses

		Maximum six-month GI toxicity	
**Variable**	**Total**, N = 162[1]	**GI-low**, N = 108[1]	**GI-high**, N = 54[1]
**Age at diagnosis (years)**	70 (67, 74)	71 (67, 74)	69 (67, 74)
**Year of treatment**	2013 (2010, 2016)	2012 (2010, 2015)	2013 (2011, 2017)
**PSA (ng/mL)**	20 (11, 33)	21 (11, 32)	18 (11, 33)
**ISUP grade group**[2]			
Group ≤ 3	53 (33%)	38 (35%)	15 (28%)
Group 4	44 (27%)	30 (28%)	14 (26%)
Group 5	60 (37%)	36 (33%)	24 (44%)
Unknown	5 (3.1%)	4 (3.7%)	1 (1.9%)
**Clinical T-stage**			
1	35 (22%)	23 (21%)	12 (22%)
2	45 (28%)	32 (30%)	13 (24%)
3	82 (51%)	53 (49%)	29 (54%)
**Number of high-risk factors**			
1	74 (46%)	50 (46%)	24 (44%)
2	67 (41%)	46 (43%)	21 (39%)
3	21 (13%)	12 (11%)	9 (17%)
**Risk group**[3]			
High	92 (57%)	62 (57%)	30 (56%)
Very high	70 (43%)	46 (43%)	24 (44%)
**Prostate volume (cc)**	51 (34, 65)	50 (33, 62)	55 (35, 70)
Unknown	12	8	4
**Elective PNRT**	110 (68%)	75 (69%)	35 (65%)
**Seminal vesicles included**	147 (91%)	98 (91%)	49 (91%)
**Radiation technique**			
3DCRT	94 (58%)	66 (61%)	28 (52%)
VMAT	68 (42%)	42 (39%)	26 (48%)
**Fiducials**	151 (93%)	100 (93%)	51 (94%)
**Antihormonal therapy**			
None	5 (3.1%)	2 (1.9%)	3 (5.6%)
Antiandrogen only	141 (87%)	94 (87%)	47 (87%)
ADT or CAB	16 (9.9%)	12 (11%)	4 (7.4%)
**Seq. of antihormonal therapy**			
Neoadjuvant only	8 (4.9%)	5 (4.6%)	3 (5.6%)
Adjuvant only	6 (3.7%)	5 (4.6%)	1 (1.9%)
Neoadjuvant and adjuvant	143 (88%)	96 (89%)	47 (87%)
None	5 (3.1%)	2 (1.9%)	3 (5.6%)
**Duration of antihormonal therapy (months)**	29 (27, 30)	29 (27, 30)	28 (28, 30)

[1] Median (IQR) or Frequency (%).

[2] According to the 2014 International Society of Urological Pathology (ISUP) Consensus Conference on Gleason Grading of Prostatic Carcinoma (9).

[3] Risk group classification adapted from Comprehensive Cancer Network (NCCN) guidelines (1).

Abbreviations: EBRT, external beam radiotherapy; GI, gastrointestinal; PSA, prostate-specific antigen; PNRT, pelvic nodal radiation therapy; 3DCRT, three-dimensional conformal radiation therapy; VMAT, volumetric modulated arc therapy; ADT, androgen deprivation therapy; CAB, combined androgen blockade, i.e., first generation antiandrogen in combination with gonadotropin-releasing hormone-agonist or antagonist.

Table A.3. Baseline characteristics for the EBRT-BT cohort, GI-analyses

		Maximum six-month GI toxicity	
**Variable**	**Total,** N = 344[1]	**GI-low**, N = 224[1]	**GI-high**, N = 120[1]
**Age at diagnosis (years)**	70 (67, 74)	70 (67, 74)	70 (66, 73)
**Year of treatment**	2016 (2013, 2018)	2016 (2013, 2018)	2016 (2013, 2018)
**PSA (ng/mL)**	19 (9, 30)	20 (9, 31)	17 (8, 30)
**ISUP grade**[2]			
Group ≤ 3	153 (44%)	99 (44%)	54 (45%)
Group 4	95 (28%)	60 (27%)	35 (29%)
Group 5	96 (28%)	65 (29%)	31 (26%)
Unknown	0 (0%)	0 (0%)	0 (0%)
**Clinical T-stage**			
1	86 (25%)	53 (24%)	33 (28%)
2	105 (31%)	65 (29%)	40 (33%)
3	153 (44%)	106 (47%)	47 (39%)
**Number of high-risk factors**			
1	208 (60%)	130 (58%)	78 (65%)
2	103 (30%)	68 (30%)	35 (29%)
3	33 (9.6%)	26 (12%)	7 (5.8%)
**Risk group**[3]			
High	254 (74%)	157 (70%)	97 (81%)
Very high	90 (26%)	67 (30%)	23 (19%)
**Prostate volume (cc)**	37 (30, 45)	35 (29, 44)	38 (30, 50)
Unknown	4	3	1
**Elective PNRT**	177 (51%)	119 (53%)	58 (48%)
**Seminal vesicles included**	306 (89%)	201 (90%)	105 (88%)
**Radiation technique**			
3DCRT	94 (27%)	60 (27%)	34 (28%)
VMAT	250 (73%)	164 (73%)	86 (72%)
**Fiducials**	67 (19%)	42 (19%)	25 (21%)
**Antihormonal therapy**			
None	15 (4.4%)	7 (3.1%)	8 (6.7%)
Antiandrogen only	278 (81%)	182 (81%)	96 (80%)
ADT or CAB	51 (15%)	35 (16%)	16 (13%)
**Seq. of antihormonal therapy**			
Neoadjuvant only	21 (6.1%)	14 (6.3%)	7 (5.8%)
Adjuvant only	1 (0.3%)	1 (0.4%)	0 (0%)
Neoadjuvant and adjuvant	307 (89%)	202 (90%)	105 (88%)
None	15 (4.4%)	7 (3.1%)	8 (6.7%)
**Duration of antihormonal therapy (months)**	28 (27, 29)	28 (27, 29)	28 (28, 29)

[1]Median (IQR) or Frequency (%).

[2]According to the 2014 International Society of Urological Pathology (ISUP) Consensus Conference on Gleason Grading of Prostatic Carcinoma (9).

[3] Risk group classification adapted from Comprehensive Cancer Network (NCCN) guidelines (1).

Abbreviations: EBRT-BT, external beam radiotherapy combined with high-dose-rate brachytherapy; GI, gastrointestinal; PSA, prostate-specific antigen; PNRT, pelvic nodal radiation therapy; 3DCRT, three-dimensional conformal radiation therapy; VMAT, volumetric modulated arc therapy; ADT, androgen deprivation therapy; CAB, combined androgen blockade, i.e., first generation antiandrogen in combination with gonadotropin-releasing hormone-agonist or antagonist.

Table A.4. Baseline characteristics for the EBRT-only cohort, grouped by baseline GU-symptom grade

Variable	Total, N = 114[1]	Baseline GU symptom grade<br>0, N = 61[1]	 1, N = 53[1]
**Age at diagnosis (years)**	69 (67, 74)	70 (67, 73)	69 (67, 74)
**Year of treatment**	2,013 (2,011, 2,017)	2,012 (2,010, 2,014)	2,014 (2,012, 2,017)
**PSA (ng/mL)**	20 (11, 32)	22 (11, 36)	17 (12, 29)
**ISUP grade[2]**			
ISUP grade ≤ 3	41 (36%)	26 (43%)	15 (28%)
ISUP grade 4	33 (29%)	15 (25%)	18 (34%)
ISUP grade 5	37 (32%)	17 (28%)	20 (38%)
Unknown	3 (2.6%)	3 (4.9%)	0 (0%)
**Clinical T-stage**			
1	31 (27%)	19 (31%)	12 (23%)
2	32 (28%)	14 (23%)	18 (34%)
3	51 (45%)	28 (46%)	23 (43%)
**Number of high-risk factors**			
1	60 (53%)	32 (52%)	28 (53%)
2	42 (37%)	23 (38%)	19 (36%)
3	12 (11%)	6 (9.8%)	6 (11%)
**Risk group[3]**			
High	72 (63%)	38 (62%)	34 (64%)
Very high	42 (37%)	23 (38%)	19 (36%)
**Prostate volume (cc)**	51 (35, 66)	53 (33, 69)	50 (36, 62)
Unknown	7	4	3
**Elective PNRT**	74 (65%)	41 (67%)	33 (62%)
**Seminal vesicles included**	103 (90%)	53 (87%)	50 (94%)
**Radiation technique**			
3DCRT	65 (57%)	43 (70%)	22 (42%)
VMAT	49 (43%)	18 (30%)	31 (58%)
**Fiducials**	105 (92%)	57 (93%)	48 (91%)
**Antihormonal therapy**			
None	4 (3.5%)	2 (3.3%)	2 (3.8%)
Antiandrogen only	100 (88%)	54 (89%)	46 (87%)
ADT or CAB	10 (8.8%)	5 (8.2%)	5 (9.4%)
**Seq. of antihormonal therapy**			
Neoadjuvant only	7 (6.1%)	4 (6.6%)	3 (5.7%)
Adjuvant only	4 (3.5%)	3 (4.9%)	1 (1.9%)
Neoadjuvant and adjuvant	99 (87%)	52 (85%)	47 (89%)
None	4 (3.5%)	2 (3.3%)	2 (3.8%)

[1]Median (IQR) or Frequency (%).

[2]According to the 2014 International Society of Urological Pathology (ISUP) Consensus Conference on Gleason Grading of Prostatic Carcinoma (9).

[3] Risk group classification adapted from Comprehensive Cancer Network (NCCN) guidelines (1).

Abbreviations: EBRT, external beam radiotherapy; GU, genitourinary; PSA, prostate-specific antigen; PNRT, pelvic nodal radiation therapy; 3DCRT, three-dimensional conformal radiation therapy; VMAT, volumetric modulated arc therapy; ADT, androgen deprivation therapy; CAB, combined androgen blockade, i.e., first generation antiandrogen in combination with gonadotropin-releasing hormone-agonist or antagonist.

Table A.5. Baseline characteristics for the EBRT-BT cohort, grouped by baseline GU-symptom grade

Variable	Total, N = 306[1]	Baseline GU symptom grade 0, N = 182[1]	1, N = 124[1]
**Age at diagnosis (years)**	70 (66, 74)	69 (67, 73)	70 (66, 74)
**Year of treatment**	2016 (2013, 2018)	2016 (2013, 2018)	2015 (2013, 2018)
**PSA (ng/mL)**	19 (9, 30)	17 (8, 29)	20 (11, 34)
**ISUP grade**[2]			
ISUP grade ≤ 3	136 (44%)	76 (42%)	60 (48%)
ISUP grade 4	86 (28%)	53 (29%)	33 (27%)
ISUP grade 5	84 (27%)	53 (29%)	31 (25%)
Unknown	0 (0%)	0 (0%)	0 (0%)
**Clinical T-stage**			
1	82 (27%)	58 (32%)	24 (19%)
2	90 (29%)	58 (32%)	32 (26%)
3	134 (44%)	66 (36%)	68 (55%)
**Number of high-risk factors**			
1	186 (61%)	125 (69%)	61 (49%)
2	91 (30%)	40 (22%)	51 (41%)
3	29 (9.5%)	17 (9.3%)	12 (9.7%)
**Risk group**[3]			
High	227 (74%)	140 (77%)	87 (70%)
Very high	79 (26%)	42 (23%)	37 (30%)
**Prostate volume (cc)**	36 (29, 45)	34 (28, 43)	39 (31, 48)
Unknown	4	3	1
**Elective PNRT**	163 (53%)	86 (47%)	77 (62%)
**Seminal vesicles included**	272 (89%)	159 (87%)	113 (91%)
**Radiation technique**			
3DCRT	92 (30%)	55 (30%)	37 (30%)
VMAT	214 (70%)	127 (70%)	87 (70%)
**Fiducials**	55 (18%)	36 (20%)	19 (15%)
**Antihormonal therapy**			
None	13 (4.2%)	12 (6.6%)	1 (0.8%)
Antiandrogen only	251 (82%)	150 (82%)	101 (81%)
ADT or CAB	42 (14%)	20 (11%)	22 (18%)
**Seq. of antihormonal therapy**			
Neoadjuvant only	19 (6.2%)	13 (7.1%)	6 (4.8%)
Adjuvant only	1 (0.3%)	1 (0.5%)	0 (0%)
Neoadjuvant and adjuvant	273 (89%)	156 (86%)	117 (94%)
None	13 (4.2%)	12 (6.6%)	1 (0.8%)

[1]Median (IQR) or Frequency (%).

[2]According to the 2014 International Society of Urological Pathology (ISUP) Consensus Conference on Gleason Grading of Prostatic Carcinoma (9).

[3] Risk group classification adapted from Comprehensive Cancer Network (NCCN) guidelines (1).

Abbreviations: EBRT-BT, external beam radiotherapy combined with high-dose-rate brachytherapy; GU, genitourinary; PSA, prostate-specific antigen; PNRT, pelvic nodal radiation therapy; 3DCRT, three-dimensional conformal radiation therapy; VMAT, volumetric modulated arc therapy; ADT, androgen deprivation therapy; CAB, combined androgen blockade, i.e., first generation antiandrogen in combination with gonadotropin-releasing hormone-agonist or antagonist.

Table A.6-A.8. Association between FFBF and GU-toxicity in the EBRT-only cohort

Multivariable Cox proportional hazard regression models

Table A.6. Main model (model 1)

Characteristic	Event N	HR[1]	95% CI[1]	p-value
Maximum six-month GU toxicity	37			
GU-low		—	—	
GU-high		2.57	1.32, 5.00	0.006
Age at Diagnosis	37	1.06	0.98, 1.15	0.2
T-stage	37			
1-2		—	—	
3		2.45	1.26, 4.74	0.008
Radiation Technique	37			
3DCRT		—	—	
VMAT		1.49	0.72, 3.09	0.3

[1]HR = Hazard Ratio, CI = Confidence Interval

Results of the main multivariable Cox proportional hazards regression analysis assessing factors associated with freedom from biochemical failure (FFBF) in the EBRT-only cohort. The model includes maximum six-month genitourinary (GU) toxicity grade, tumour stage (T-stage), and radiation technique.

Sensitivity analyses

Table A.7. Model 2

Characteristic	Event N	HR[1]	95% CI[1]	p-value
Maximum six-month GU toxicity	37			
GU-low		—	—	
GU-high		2.83	1.44, 5.56	0.002
Age at Diagnosis	37	1.08	1.00, 1.17	0.065
T-stage	37			
1-2		—	—	
3		2.25	1.14, 4.42	0.019
Elective PNRT	37			
No		—	—	
Yes		1.69	0.66, 4.33	0.3
ISUP-grade	37			
ISUP grade ≤ 3		—	—	
ISUP grade 4		1.34	0.60, 2.98	0.5
ISUP grade 5		1.63	0.70, 3.79	0.3

[1]HR = Hazard Ratio, CI = Confidence Interval

Results of the alternative multivariable Cox regression model assessing factors associated with freedom from biochemical failure (FFBF) in the EBRT-only cohort, including maximum six-month genitourinary (GU) toxicity grade, tumour stage (T-stage), addition of elective pelvic nodal radiotherapy (PNRT) and ISUP-grade.

Table A.8. Association between FFBF and baseline GU-symptom grade

Characteristic	Event N	HR[1]	95% CI[1]	p-value
Baseline GU grade	37			
0		—	—	
1		1.51	0.75, 3.03	0.3
Age at Diagnosis	37	1.05	0.97, 1.13	0.3
T-stage	37			
1-2		—	—	
3		2.38	1.22, 4.61	0.010
Radiation Technique	37			
3DCRT		—	—	
VMAT		1.35	0.62, 2.94	0.4

[1]HR = Hazard Ratio, CI = Confidence Interval

Results of a multivariable Cox proportional hazards regression analysis assessing factors associated with freedom from biochemical failure (FFBF) in the EBRT-only cohort. The model includes baseline genitourinary (GU) symptom grade, tumour stage (T-stage), and radiation technique.

Table A.9-A.10. Association between FFBF and GI-toxicity in the EBRT-only cohort

Cox proportional hazard regression models

Table A.9. Main model (model 1)

Characteristic	Event N	HR[1]	95% CI[1]	p-value
Maximum six-month GI toxicity	58			
GI-low		—	—	
GI-high		1.52	0.89, 2.59	0.12
Age at Diagnosis	58	1.00	0.94, 1.06	>0.9
T-stage	58			
1-2		—	—	
3		2.63	1.52, 4.58	<0.001
Radiation Technique	58			
3DCRT		—	—	
VMAT		1.66	0.93, 2.95	0.084

[1]HR = Hazard Ratio, CI = Confidence Interval

Results of the main multivariable Cox proportional hazards regression analysis assessing factors associated with freedom from biochemical failure (FFBF) in the EBRT-only cohort. The model includes maximum six-month gastrointestinal (GI) toxicity grade, tumour stage (T-stage), and radiation technique.

Sensitivity analyses

Table A.10. Model 2

Characteristic	Event N	HR[1]	95% CI[1]	p-value
Maximum six-month GI toxicity	58			
GI-low		—	—	
GI-high		1.53	0.90, 2.60	0.12
Age at Diagnosis	58	1.00	0.94, 1.07	>0.9
T-stage	58			
1-2		—	—	
3		2.59	1.48, 4.55	<0.001
Radiation Volume	58			
PORT		—	—	
WPRT		1.05	0.50, 2.20	0.9
ISUP-grade	58			
ISUP grade ≤ 3		—	—	
ISUP grade 4		1.66	0.84, 3.26	0.14
ISUP grade 5		2.07	1.07, 4.02	0.030

[1]HR = Hazard Ratio, CI = Confidence Interval

Results of the alternative multivariable Cox regression model assessing factors associated with freedom from biochemical failure (FFBF) in the EBRT-only cohort, including maximum six-month gastrointestinal (GI) toxicity grade, tumour stage (T-stage), addition of elective pelvic nodal radiotherapy (PNRT) and ISUP-grade.

Table A.11-A.15. Association between FFBF and GU-toxicity in the EBRT-BT cohort

Cox proportional hazard regression models

Table A.11. Main model (model 1)

Characteristic	Event N	HR[1]	95% CI[1]	p-value
Maximum six-month GU toxicity	64			
GU-low		—	—	
GU-high		1.05	0.63, 1.75	0.8
Age at Diagnosis	64	0.99	0.95, 1.05	0.8
T-stage	64			
1-2		—	—	
3		1.04	0.64, 1.70	0.9
Radiation Technique	64			
3DCRT		—	—	
VMAT		2.62	1.41, 4.86	0.002

[1]HR = Hazard Ratio, CI = Confidence Interval

Results of the main multivariable Cox proportional hazards regression analysis assessing factors associated with freedom from biochemical failure (FFBF) in the EBRT-BT cohort. The model includes maximum six-month genitourinary (GU) toxicity grade, tumour stage (T-stage), and radiation technique.

Sensitivity analyses

Table A.12. Model 2

Characteristic	Event N	HR[1]	95% CI[1]	p-value
Maximum six-month GU toxicity	64			
GU-low		—	—	
GU-high		1.14	0.68, 1.89	0.6
Age at Diagnosis	64	0.99	0.94, 1.04	0.7
T-stage	64			
1-2		—	—	
3		1.16	0.70, 1.93	0.6
Elective PNRT	64			
No		—	—	
Yes		1.22	0.69, 2.14	0.5
ISUP-grade	64			
ISUP grade ≤ 3		—	—	
ISUP grade 4		1.60	0.81, 3.14	0.2
ISUP grade 5		3.80	2.06, 7.02	<0.001

[1]HR = Hazard Ratio, CI = Confidence Interval

Results of the alternative multivariable Cox regression model assessing factors associated with freedom from biochemical failure (FFBF) in the EBRT-BT cohort, including maximum six-month genitourinary (GU) toxicity grade, tumour stage (T-stage), addition of elective pelvic nodal radiotherapy (PNRT) and ISUP-grade.

Table A.13. Association between FFBF and baseline GU-symptom grade

Characteristic	Event N	HR[1]	95% CI[1]	p-value
Baseline GU grade	64			
0		—	—	
1		2.34	1.41, 3.90	0.001
Age at Diagnosis	64	0.99	0.94, 1.04	0.7
T-stage	64			
1-2		—	—	
3		0.89	0.54, 1.47	0.6
Radiation Technique	64			
3DCRT		—	—	
VMAT		2.59	1.40, 4.78	0.002

[1]HR = Hazard Ratio, CI = Confidence Interval

Results of a multivariable Cox proportional hazards regression analysis assessing factors associated with freedom from biochemical failure (FFBF) in the EBRT-BT cohort. The model includes baseline genitourinary (GU) symptom grade, tumour stage (T-stage), and radiation technique.

Subgroup analyses, stratified by baseline GU toxicity grade

Table A.14. Baseline GU symptom grade 0

Characteristic	Event N	HR[1]	95% CI[1]	p-value
Maximum six-month GU toxicity	26			
GU-low		—	—	
GU-high		0.42	0.14, 1.24	0.12
Age at Diagnosis	26	0.97	0.90, 1.04	0.4
T-stage	26			
1-2		—	—	
3		1.32	0.61, 2.86	0.5
Radiation Technique	26			
3DCRT		—	—	
VMAT		2.41	0.95, 6.12	0.065

[1]HR = Hazard Ratio, CI = Confidence Interval

Results of multivariable Cox proportional hazards regression analysis assessing factors associated with freedom from biochemical failure in a subgroup of the EBRT-BT cohort, including patients with baseline GU symptom grade 0. The model includes maximum six-month genitourinary (GU) toxicity grade, tumour stage (T-stage), and radiation technique.

Table A.15. Baseline GU symptom grade 1

**Characteristic**	**Event N**	**HR[1]**	**95% CI[1]**	**p-value**
Maximum six-month GU toxicity	38			
GU-low		—	—	
GU-high		1.27	0.67, 2.43	0.5
Age at Diagnosis	38	1.01	0.94, 1.08	0.8
T-stage	38			
1-2		—	—	
3		0.67	0.36, 1.28	0.2
Radiation Technique	38			
3DCRT		—	—	
VMAT		2.86	1.24, 6.58	0.013

[1]HR = Hazard Ratio, CI = Confidence Interval

Results of multivariable Cox proportional hazards regression analysis assessing factors associated with freedom from biochemical failure in a subgroup of the EBRT-BT cohort, including patients with baseline GU symptom grade 1. The model includes maximum six-month genitourinary (GU) toxicity grade, tumour stage (T-stage), and radiation technique.

Table A.16-A.17. Association between FFBF and GI-toxicity in the EBRT-BT cohort

Cox proportional hazard regression models

Table A.16. Main model (model 1)

Characteristic	Event N	HR[1]	95% CI[1]	p-value
Maximum six-month GI toxicity	72			
GI-low		—	—	
GI-high		1.11	0.68, 1.79	0.7
Age at Diagnosis	72	1.00	0.95, 1.05	>0.9
T-stage	72			
1-2		—	—	
3		1.18	0.75, 1.88	0.5
Radiation Technique	72			
3DCRT		—	—	
VMAT		2.69	1.47, 4.94	0.001

[1]HR = Hazard Ratio, CI = Confidence Interval

Results of the main multivariable Cox proportional hazards regression analysis assessing factors associated with freedom from biochemical failure (FFBF) in the EBRT-BT cohort. The model includes maximum six-month gastrointestinal (GI) toxicity grade, tumour stage (T-stage), and radiation technique.

Sensitivity analyses

Table A.17. Model 2

Characteristic	Event N	HR[1]	95% CI[1]	p-value
Maximum six-month GI toxicity	72			
GI-low		—	—	
GI-high		1.14	0.70, 1.86	0.6
Age at Diagnosis	72	0.99	0.94, 1.04	0.7
T-stage	72			
1-2		—	—	
3		1.34	0.83, 2.18	0.2
Elective PNRT	72			
No		—	—	
Yes		1.24	0.74, 2.10	0.4
ISUP-grade	72			
ISUP grade ≤ 3		—	—	
ISUP grade 4		1.81	0.98, 3.36	0.059
ISUP grade 5		3.38	1.89, 6.05	<0.001

[1]HR = Hazard Ratio, CI = Confidence Interval

Results of the alternative multivariable Cox regression model assessing factors associated with freedom from biochemical failure (FFBF) in the EBRT-BT cohort, including maximum six-month gastrointestinal (GI) toxicity grade, tumour stage (T-stage), addition of elective pelvic nodal radiotherapy (PNRT) and ISUP-grade.

Table A.18-A.19. Association between MFS and GU-toxicity in the EBRT-only cohort

Table A.18. Cox proportional hazard regression

Characteristic	Event N	HR[1]	95% CI[1]	p-value
Maximum six-month GU toxicity	45			
GU-low		—	—	
GU-high		2.22	1.21, 4.07	0.010
Age at Diagnosis	45	1.08	1.00, 1.16	0.044
T-stage	45			
1-2		—	—	
3		1.50	0.83, 2.71	0.2
Radiation Technique	45			
3DCRT		—	—	
VMAT		1.35	0.69, 2.64	0.4

[1]HR = Hazard Ratio, CI = Confidence Interval

Results of multivariable Cox proportional hazards regression analysis assessing factors associated with metastasis free survival (MFS), defined as the absence of any of the following: regional or distant metastasis, or death, in the EBRT-only cohort. The model includes maximum six-month genitourinary (GU) toxicity grade, tumour stage (T-stage), and radiation technique.

Table A.19. Fine-Gray regression

Characteristic	HR[1]	95% CI[1]	p-value
Maximum six-month GU toxicity			
GU-low	—	—	
GU-high	2.66	1.27, 5.60	0.010
Age	1.04	0.94, 1.15	0.5
T-stage			
1-2	—	—	
3	3.53	1.72, 7.26	<0.001
Radiation technique			
3DCRT	—	—	
VMAT	1.70	0.79, 3.67	0.2

[1]HR = Hazard Ratio, CI = Confidence Interval

Subdistribution hazard ratios for metastasis free survival (MFS), estimated using the Fine-Gray regression model, accounting for death from any cause as competing risk event, in the EBRT-only cohort. The model includes maximum six-month genitourinary (GU) toxicity grade, tumour stage (T-stage), and radiation technique.

Table A.20. Association between PCSM and GU-toxicity in the EBRT-only cohort

A.20. Fine-Gray regression

Characteristic	HR[1]	95% CI[1]	p-value
Maximum six-month GU toxicity			
GU-low	—	—	
GU-high	2.35	0.90, 6.15	0.082
Age	1.09	0.98, 1.22	0.10
T-stage			
1-2	—	—	
3	4.49	1.67, 12.0	0.003
Radiation technique			
3DCRT	—	—	
VMAT	1.27	0.44, 3.71	0.7

[1]HR = Hazard Ratio, CI = Confidence Interval

Subdistribution hazard ratios for prostate cancer specific mortality (PCSM), estimated using the Fine-Gray regression model, accounting for death from other causes as competing risk event, in the EBRT-only cohort. The model includes maximum six-month genitourinary (GU) toxicity grade, tumour stage (T-stage), and radiation technique.

Figure A.1 Estimated cumulative incidence of metastatic disease, low versus high GU-toxicity grade, in the EBRT-only and EBRT-BT cohort

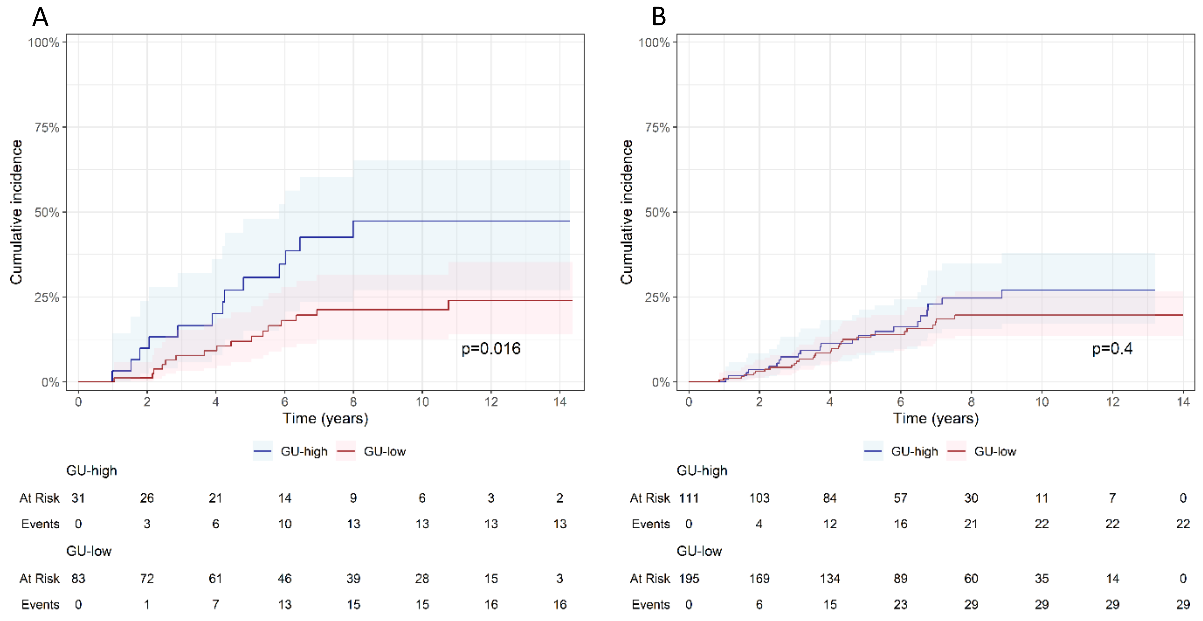

Cumulative incidence curve depicting the proportion of patients experiencing regional or distant metastases, with death from any cause as competing event, comparing high grade (blue line) and low grade (red line) maximum six-month GU toxicity in the EBRT-only (A) and EBRT-BT (B) cohort. Shaded areas around the curves represent 95% confidence intervals. P-values calculated using Gray's test.

Abbreviations: GU, genitourinary; EBRT, external beam radiotherapy; BT, brachytherapy.

Figure A.2 Estimated cumulative incidence of prostate-cancer specific death, low versus high GU-toxicity grade, in the EBRT-only and EBRT-BT cohort

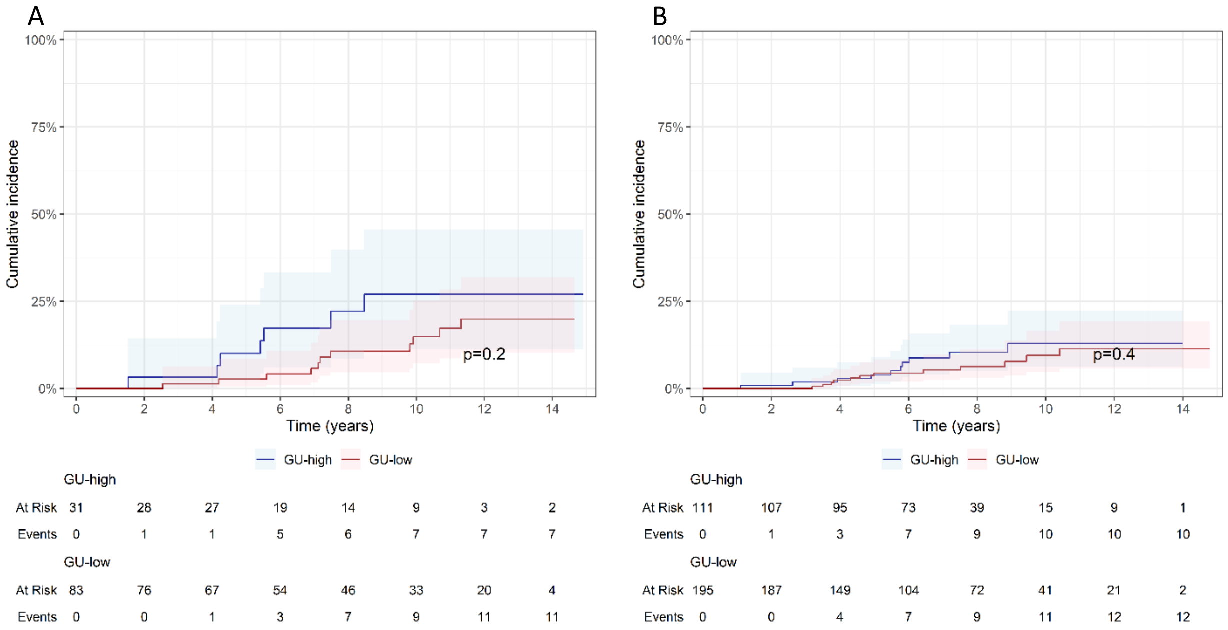

Cumulative incidence curve depicting the proportion of patients experiencing prostate cancer specific death, with death from other causes as competing event, comparing high grade (blue line) and low grade (red line) maximum six-month GU toxicity in the EBRT-only (A) and EBRT-BT (B) cohort. Shaded areas around the curves represent 95% confidence intervals. P-values calculated using Gray's test.

Abbreviations: GU, genitourinary; EBRT, external beam radiotherapy; BT, brachytherapy.

References

[1] Cox JD, Stetz J, Pajak TF. Toxicity criteria of the Radiation Therapy Oncology Group (RTOG) and the European Organization for Research and Treatment of Cancer (EORTC). Int J Radiat Oncol Biol Phys. 1995;31(5):1341-6.

[2] Epstein JI, Egevad L, Amin MB, Delahunt B, Srigley JR, Humphrey PA. The 2014 International Society of Urological Pathology (ISUP) Consensus Conference on Gleason Grading of Prostatic Carcinoma: Definition of Grading Patterns and Proposal for a New Grading System. Am J Surg Pathol. 2016;40(2):244-52.

[3] National Comprehensive Cancer Network. Prostate Cancer (Version 3.2024) [Available from: https://www.nccn.org/professionals/physician_gls/pdf/prostate.pdf.
